# Dependence of nighttime sleep duration in one-month-old infants on alterations in natural and artificial photoperiod

**DOI:** 10.1038/srep44749

**Published:** 2017-03-17

**Authors:** Sachiko Iwata, Fumie Fujita, Masahiro Kinoshita, Mitsuaki Unno, Takashi Horinouchi, Seiichi Morokuma, Osuke Iwata

**Affiliations:** 1 Centre for Developmental and Cognitive Neuroscience, Department of Paediatrics and Child Health, Kurume University School of Medicine, Fukuoka, Japan; 2 Kurume University School of Nursing, Fukuoka, Japan; 3 Department of Obstetrics and Gynaecology, Kurume University School of Medicine, Fukuoka, Japan; 4 Department of Obstetrics and Gynaecology, Kyushu University Hospital, Fukuoka, Japan

## Abstract

Human sleep–wake cycles are entrained by both natural and artificial light–dark cycles. However, little is known regarding when and how the photoperiod changes entrain the biological clock after conception. To investigate the dependence of sleep patterns in young infants on the natural and artificial light–dark cycles, 1,302 pairs of one-month-old infants and their mothers were asked to answer a questionnaire. Birth in spring, longer daytime sleep duration, early/regular light-off times, and longer maternal nighttime sleep duration were identified as independent variables for longer infant nighttime sleep duration in both univariate and multivariate analyses. Longer maternal nighttime sleep duration was dependent on shorter naps and early/regular bed times but not on the season. We found that nighttime sleep duration depended on both natural and artificial diurnal photoperiod changes in one-month-old infants. Although sleep patterns of infants mimicked those of their mothers, nighttime sleep duration depended on the season, and was positively associated with daytime sleep duration, only in the infants. These specific variables, which render sleep patterns of the infants different from those of their mothers, might be a clue to reveal the covert acquisition process of mature circadian rhythms after birth.

Light is the principal extrinsic zeitgeber, which helps the biological clock synchronise with environmental cycles^1^,^2^. Light information is transmitted from retinal photoreceptor cells to the suprachiasmatic nucleus (SCN) of hypothalamus, which functions as a master clock for biological rhythms in mammals^2^,^3^. These phototransduction components are differentiated in utero and start generating rudimentary circadian rhythms even before being directly entrained by light^2^. Entrainment of physiological and behavioural rhythms to external light cycles overlaps with the maturation of individual phototransduction elements^2^. As the pineal production of melatonin is inhibited by light, plasma melatonin levels follow the changes in photoperiods, causing seasonal biological changes in the sleep–wake rhythm and thermoregulatory cycle in various organisms^4^,^5^,^6^. Relationships among photoperiod, melatonin production, and sleep have already been established in controlled conditions and have determined that shorter photoperiods are associated with longer sleep periods in adult species^7^,^8^,^9^.

Sleep in newborn infants is distributed throughout the day and night. Diurnal sleep rhythms begin to emerge at around 10–12 weeks after birth, when the sleep of infants becomes increasingly nocturnal^10^. Sleep problems of young infants are common, as more than 25% of Australian parents reported sleep problems of their infants in the first 6 months of life^11^. Sleep problems during infancy are associated with increase in maternal depression, anxiety and fatigue^12^,^13^. Sleep difficulty and other regulatory problems of infants are related with their lower intelligence quotient, behavioural problems, attention and hyperactivity problems at school age^14^. Understanding of the acquisition of nocturnal sleep rhythms after birth may help improve mental and physical condition of mothers of young infants, and may help develop strategies to prevent, or at least predict later developmental problems. To date, however, the actual maturation process of human sleep following birth has not fully been elucidated; no published information exists regarding the influence of seasonal photoperiod changes on the sleep patterns of young infants. Given that the daily lighting patterns are artificially controlled in the modern world, changes in the natural photoperiod may not have significant influence on the sleep pattern of young infants.

To assess the dependence of nighttime sleep duration in young infants and their mothers on natural and artificial photoperiod changes, we performed a cross-sectional study over 1 year in a large population of one-month-old infants and their mothers.

## Results

Of the 1,302 questionnaires filled out by the participants, 227 were excluded because the questionnaires were missing more than 10% of the data (*n* = 104), lacked essential variables or both (*n* = 123). Subsequently, data were analysed for the remaining 1,075 pairs of infants and mothers.

### Study population characteristics

Fourteen infants (1.3%) were born prematurely, between 35 and 36 weeks’ gestation, but did not require any special medical care. Most mothers were satisfied with their household income (92%) and were high school graduates or had advanced academic degrees (97%), suggesting that the participating mothers were mostly middle class (Table 1).

### Infant and maternal sleep patterns

**Figure 1:**
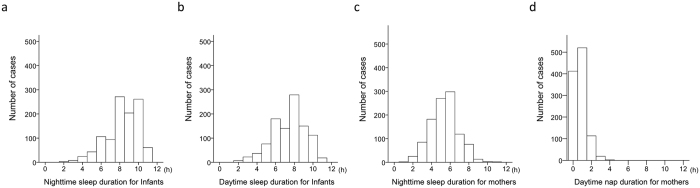
Distribution of nighttime and daytime sleep duration. Histograms depicting sleep durations of infants (**a**,**b**) and their mothers (**c**,**d**) during the nighttime (20:00 to 8:00 h; **a** and **c**) and daytime (8:00 to 20:00 h; **b** and **d**).

Means and standard deviation (SD) for daytime and nighttime sleep duration in infants were 7.4 (1.8) and 8.3 (1.8) h, respectively, indicating infants slept significantly longer at night (p < 0.001, Fig. 1). Nighttime sleep duration in mothers was 5.5 (1.5) h. A total of 669 mothers regularly napped during the day for 1.0 (0.6) h.

### Dependence of variables on the season

Gestational age, birth weight, whether mothers breastfed, maternal education, and satisfaction with familial income were independent of the infant’s birth season, although more first babies were born in autumn (p = 0.007; Table 1). No seasonal difference was found in the incidence of regular and/or early light-off times for infants or maternal bed times.

### Variables affecting infant nighttime sleep duration

**Figure 2:**
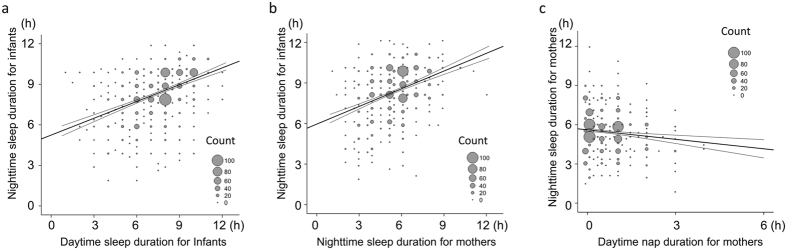
Relationships between nighttime and daytime sleep durations of infants and their mothers. Binned scatter plots with regression lines and their 95% confidence intervals depicting relationships between nighttime and daytime sleep durations of infants (**a**), nighttime sleep durations of infants and their mothers (**b**), and nighttime and daytime sleep durations of mothers (**c**). Nighttime sleep duration in infants was positively associated with both their daytime sleep duration (**a**; p < 0.001) and maternal nighttime sleep duration (**b**; p < 0.001), whereas longer maternal daytime naps were negatively associated with their nighttime sleep duration (**c**; p < 0.001).

**Figure 3:**
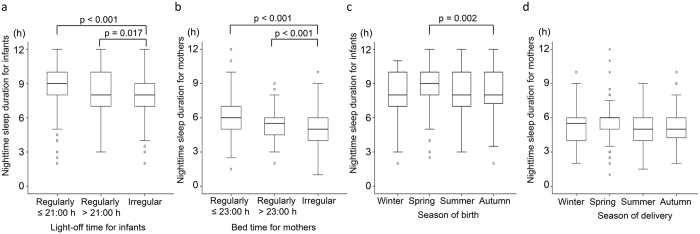
Dependence of nighttime sleep durations on the season and sleep schedule. Box plots depicting the dependence of nighttime sleep durations on the light-off/bed time (**a**,**b**) and season of birth/delivery (**c**,**d**) for infants (**a** and **c**) and their mothers (**b** and **d**). Nighttime sleep duration in infants with irregular light-off times was shorter than those with regular early (p < 0.001) or regular late (p = 0.017) light-off times (**a**). Nighttime sleep duration for mothers with irregular bed times was shorter than that for those with regular early or regular late bed times (**b**; both p < 0.001). The duration of nighttime sleep in infants born in spring was longer than those born in autumn (**c**; p = 0.002), whereas no seasonal difference was found in maternal nighttime sleep duration (**d**). Solid lines, median; boxes, lower and upper quartiles; whiskers, minimum and maximum; circles, outliers; stars, extreme outliers.

Nighttime sleep duration in infants was positively associated with both their daytime sleep duration and maternal nighttime sleep duration (both p < 0.001) (Table 2 and Fig. 2). The duration of nighttime sleep in infants with irregular light-off times was shorter than those with regular early or regular late light-off times (p < 0.001 and p = 0.017, respectively, Fig. 3). The brightness of the infant’s bedroom during the day did not affect the nighttime sleep duration in infants. Infants born in spring slept longer at night than those born in autumn (p = 0.002). A multivariate analysis showed that infant nighttime sleep duration was dependent on the season of birth, irregular light-off times, infant daytime sleep duration, and maternal nighttime sleep duration (Table 2).

### Variables affecting maternal nighttime sleep duration

In contrast to our finding in infants, no seasonal difference was found in maternal nighttime sleep duration (Supplemental Table 1 and Fig. 3). However, longer maternal daytime naps were negatively associated with nighttime sleep duration in mothers (p < 0.001, Fig. 2). Nighttime sleep duration for mothers with irregular bed times was shorter than that for those with regular early or regular late bed times (both p < 0.001, Fig. 3).

## Discussion

This survey study investigated potential independent variables associated with the sleep patterns of one-month-old infants and their mothers. One overarching goal of our work is to determine how steady acquisition of nocturnal sleep patterns occurs in newborn infants to subsequently develop strategies for improving the quality of life in mothers with young infants. Our results showed that nighttime sleep duration in one-month-old infants was associated with the season of birth as well as the timing and regularity of the light-off time. This is, to our knowledge, the first study to suggest the dependence of young infants’ sleep on both natural and artificial photoperiod changes.

### Circadian rhythms in foetuses and its preservation after birth

Despite the lack of overt diurnal rhythms in newborn infants, studies in humans and nonhuman primates have revealed immature but clear 24-h rhythms in foetal heart rate and respiratory movements during the latter half of pregnancy^15^,^16^,^17^. Maternal melatonin, which readily crosses the placenta unaltered, plays a major role in the modulation of the foetal clock^18^,^19^. Nighttime melatonin levels in pregnant women increase after 24 weeks of gestation and reach their maximum level at term^20^,^21^, suggesting that foetuses are exposed to a relatively high level of melatonin during the final weeks of gestation. Maternal exposure to an alternating photoperiodic environment changes the rhythmic expression of foetal clock genes via melatonin receptors in the foetal SCN, and this expression can be reversed when daily melatonin injections are given to the mother^22^,^23^,^24^. Thus, maternal melatonin is likely to provide photoperiodic information to the foetus, and, hence, the foetal circadian system may be sensitive to changes in extra-uterine environments.

Studies in animals suggest that maternal coordination of the foetal circadian photic and melatonin responses in utero influences the postnatal rhythm system^25^,^26^,^27^, although its regulation has not been described in detail. Disturbances in the maternal circadian system have long-term consequences on behavioural and metabolic functions in the offspring, highlighting the persistent and extensive impact of maternal entrainment on the foetal circadian clock^28^,^29^. In our recent study focused on the diurnal cortisol rhythm after birth in preterm and term infants, we found that cortisol levels are higher in the evening than in the morning up to 8 weeks of age, suggesting that the foetal circadian clock entrained in utero may persistently influence the adrenal rhythm after birth^30^,^31^. In addition, Sivan and colleagues reported that a urinary melatonin analogue in infants is elevated in June and declines in December up to 8 weeks after birth^32^. Considering these results along with those in our present study, longer nighttime sleep duration of infants born in spring may be explained by their exposure to relatively higher circulating melatonin levels during the last weeks of gestation as well as their own subsequently produced high serum melatonin levels.

### Influence of the light–dark cycle on sleep patterns

Light deprivation leads to an elevation of plasma melatonin levels, even in newborn infants when their eyes are masked during phototherapy for hyperbilirubinemia^33^. However, studies in both animals and humans have suggested that exposure to light is also essential for the development of the circadian system after birth^1^,^2^. While melatonin secretion is attenuated during light exposure, sufficient daylight promotes the production of serotonin, the precursor of melatonin^34^,^35^. Continuous lighting for newborn infants has negative effects on the establishment of circadian rhythms^1^,^36^, confirming the importance of the light–dark cycle for the acquisition of mature diurnal cycles even for newborn infants. In the current study, early light-off times were associated with longer nighttime sleep duration, suggesting that light–dark cycles affect sleep patterns of infants even one month after birth. In addition to an early light-off time, we found that late but regular light-off times were associated with long nighttime sleep duration in infants, suggesting that regular light–dark cycles are more efficient than irregular cycles in the entrainment of the immature circadian clock. When regular early light-off times are difficult to achieve, late but regular light-off times may still help secure a sufficient duration of nighttime sleep in young infants and their families.

### Synchrony of sleep cycles between mothers and infants

In the current study, infant and mother nighttime sleep duration was tightly linked. The sleep patterns of mothers are inevitably affected by those of their infants, as feeding is involved in the sleep–wake cycle of infants. Although the infants’ sleep patterns mimicked those of their mothers, the relationship between daytime and nighttime sleep duration differed in infants and mothers. Longer daytime napping in the mothers was associated with shorter nighttime sleep. This finding is consistent to previous reports, which highlighted the negative effect of excessive naps on nighttime sleep in children and adults^37^,^38^. Obtaining good night sleep after long naps would be difficult even for mothers who are under chronic sleep deprivation. However, nighttime sleep deprivation may in turn have increased the demand for catch-up sleep during the day. We are currently undertaking a study examining maternal depression scores and sleep satisfaction to determine the benefit and harm of daytime naps in mothers of young infants.

It was interesting that, in infants, longer daytime sleep was positively correlated with nighttime sleep duration. Several explanations are possible. As previously discussed, infants with relatively higher circulating melatonin levels might sleep longer regardless of day and night. Several specific family lifestyles, maternal habits and other environmental conditions might help promote relatively sounder and longer sleep throughout the day. Studies using non-invasive salivary biomarkers and actigraphy may help determine the exact mechanism of the observed associations.

### Limitations of the study

This survey study was based on questionnaire answers provided by mothers of one-month-old infants. Therefore, unlike previous studies that have used objective biomarkers, such as those obtain using actigraphy and salivary markers, the reliability of the results depended on the quality of the observations by the mothers. Statistical findings from univariate analysis were not corrected for multiple comparisons because of the exploratory nature of our study. Therefore, caution is required to translate the contribution of several independent variables, which showed only modest correlations with sleep patterns. To assess the influence of the birth season on the sleep patterns of infants, we conducted the study over one year. However, the parity of infants was dependent on the season, with uniparae being most common in autumn. The dependence of parity on the season has been recognised previously, the pattern of which alters according to the social and temporal background of the region^39^,^40^. We found seasonality to be a significant variable in infant nighttime sleep duration even after adjustment for parity (data not shown), suggesting that any influence of season-specific parity is likely to be minimal. Finally, we speculated that the season-specific photoperiod was informed to the circadian clock of foetuses in utero, whereas the diurnal light–dark cycle of the post-natal environment entrained the circadian clock of newborn infants. However, we did not provide direct evidence to support this hypothesis. Future longitudinal studies will be required to elucidate the timing of entrainment using non-invasive biomarkers in mothers, foetuses, and newborn infants.

## Conclusions

Here, we found that the duration of nighttime sleep in one-month-old infants depended on both seasonal and artificial photoperiod changes. Infants born in spring and those exposed to regular or early light-off times were associated with longer nighttime sleep duration. Although a strong mother–infant relationship was observed in their sleep patterns, nighttime sleep duration was dependent on the seasonal sleep variation, and was positively associated with daytime sleep duration, only in the infants. These specific variables, which render sleep patterns of the infants different from those of their mothers, might provide a clue to elucidate the covert acquisition process of nocturnal sleep patterns after birth. Further studies need to address when and how the season-specific photoperiod and the diurnal light–dark cycle influence the circadian clock of foetuses and newborn infants.

## Methods

### Ethics statement

This study was conducted at four obstetrics hospitals in Fukuoka prefecture in compliance with the Declaration of Helsinki, and with the approval of the ethics committees of Kurume University School of Medicine and Kyushu University School of Medicine. Informed consent was obtained from all respondents of the questionnaires.

### Study population

Fukuoka prefecture has a population of 5,104,197 and 46,005 annual births. Daylight saving time is not observed in Japan. Located at latitude 33.3°N, in the southern part of Japan, Fukuoka shows a clear seasonal difference in photoperiod, with 14.4 h of light in the summer compared with only 10.0 h in winter. Two tertiary perinatal centres (Kurume University Hospital and Kyushu University Hospital) and three private obstetrics hospitals (Izumi Ladies’ Clinic, Fukuoka Birth Clinic and Sato Women’s Hospital) located in Fukuoka prefecture participated in this study.

Mothers who brought their infants to their one-month baby check between December 2014 and November 2015 were asked to answer a questionnaire (Supplemental Text 1). Mothers whose infants required hospitalisation before the baby check were not recruited. The study participant was asked to answer a questionnaire to provide information about the infant (variables at birth, feeding methods, brightness of the infant’s room, and sleep status within 7 days of the visit), the mother (educational background, daily life schedule, social environment, satisfaction with the household income, sleep patterns, and mental condition within 7 days of the visit) and the family (family structure). Nighttime sleep duration for the infant was defined as the time spent sleeping between 20:00 and 8:00 h regardless of the season. For infants with regular light-off times, the typical light-off time was asked to discriminate late light-off times, that is, after 21:00 h. Similarly, for mothers whose bed times were regular, the typical bed time was asked to identify late bed times, that is after 23:00 h. These thresholds were set to the median light-off time for infants (21:00 h) and bed time for adults (23:00 h) reported in national surveys^41^,^42^.

### Data and statistical analyses

Questionnaires missing greater than 10% of the requested data without relevant reasons, or those lacking any essential sleep variable (i.e., daytime/nighttime sleep durations of the infant and/or mother) were excluded from the analysis. To assess the dependence of sleep variables on the birth season, data were binned into groups of four seasons based on the birth month: winter (from December to February), spring (from March to May), summer (from June to August) and autumn (from September to November). Seasonal differences in potential independent variables for infant and mother nighttime sleep duration were assessed using an analysis of variance for continuous variables and the chi-square test for nominal variables. Independent variables for infant and mother nighttime sleep duration, and the potential interaction of the sleep duration between the infant and mother, were assessed using a generalised linear model.

## Additional Information

**How to cite this article:** Iwata, S. *et al*. Dependence of nighttime sleep duration in one-month-old infants on alterations in natural and artificial photoperiod. *Sci. Rep.* **7**, 44749; doi: 10.1038/srep44749 (2017).

**Publisher's note:** Springer Nature remains neutral with regard to jurisdictional claims in published maps and institutional affiliations.

## Supplementary Material

Supplemental Information

## Figures and Tables

**Table 1:** Participant characteristics and their dependence on the birth season.

Variable	n	(%)	p^‡^
Familial background			
Paternal age (year)^†^	33.5	5.3	0.321
Maternal age (year)^†^	31.9	4.6	0.176
Maternal education (4 missing)			
Junior high school	32	3.0	0.678
High school	247	23.1	
College	792	73.9	
Satisfaction with family income (15 missing)			
Fully satisfied	167	15.8	0.413
Generally satisfied	806	76.0	
Not satisfied	87	8.2	
Information at birth (52 missing)			
Gestational age (week)^†^	39.5	1.2	0.692
Birth weight (g)^†^	3089	371	0.440
Season			
Winter	231	21.5	NA
Spring	311	28.9	
Summer	237	22.0	
Autumn	296	27.5	
Parity^††^ (52 missing)			
1	432	42.2	0.007
2	385	37.6	
≥3	206	20.1	
Lifestyle			
Primary feeding method (7 missing)			
Breast milk	864	80.9	0.126
Formula	204	19.1	
Room brightness (2 missing)			
Bright	815	76.0	0.906
Dim	258	24.0	
Light-off time for infants (6 missing)			0.135
Regularly ≤ 21:00 h	364	34.1	
Regularly > 21:00 h	478	44.7	
Irregular	227	21.2	
Maternal bed time			
Regularly ≤ 23:00 h	392	36.5	0.651
Regularly > 23:00 h	162	15.1	
Irregular	521	48.5	

^†^Data are presented as mean and standard deviation, rather than as number and percentage. ^††^Parity includes the current delivery. ^‡^Dependence of clinical variables on the season was assessed using analysis of variance or chi-square test where relevant. NA, not applicable.

**Table 2:** Control variables of nighttime sleep duration for infants.

	β	95% confidence interval		p
		Lower	Upper	
Univariate analysis				
Season				
Winter	1.11	0.83	1.50	0.490
Spring	1.54	1.17	2.03	0.002
Summer	1.16	0.87	1.55	0.325
Autumn	Reference			
Room brightness				
Bright	1.14	0.89	1.47	0.303
Dim	Reference			
Light-off time for infants				
Regularly ≤ 21:00 h	2.19	1.62	2.96	<0.001
Regularly >21:00 h	1.42	1.06	1.89	0.017
Irregular	Reference			
Daytime sleep duration for infants (h)	1.51	**1.41**	1.61	<0.001
Maternal nighttime sleep duration (h)	1.57	**1.44**	1.66	<0.001
Multivariate analysis				
Season				
Winter	1.11	0.86	1.43	0.418
Spring	1.37	1.07	1.75	0.011
Summer	1.22	0.96	1.54	0.098
Autumn	Reference			
Light-off time for infants				
Regularly ≤ 21:00 h	1.83	1.42	2.35	<0.001
Regularly > 21:00 h	1.36	1.08	1.72	0.010
Irregular	Reference			
Daytime sleep duration for infants (h)	1.46	1.38	1.54	<0.001
Maternal nighttime sleep duration (h)	1.45	1.36	1.55	<0.001

Independent variables for infant nighttime sleep duration were assessed using a generalised linear model.
