# Supplementary material for: Dependence of nighttime sleep duration in one-month-old infants on alterations in natural and artificial photoperiod
Source: Sci Rep. 2017 Mar 17;7:44749. doi: 10.1038/srep44749 (PMC5355994; doi:10.1038/srep44749)
Supplement: Supplemental Information [file srep44749-s1.doc]

**Online Supplemental Information**

**Title:**

**Dependence of nighttime sleep duration in one-month-old infants on alterations in natural and artificial photoperiod**

**Authors:**

Sachiko Iwata^1^, Fumie Fujita^2^, Masahiro Kinoshita^1^, Mitsuaki Unno^1^, Takashi Horinouchi^3^, Seiichi Morokuma^4^ and Osuke Iwata^1^.

**Affiliations:**

1. Centre for Developmental and Cognitive Neuroscience, Dept of Paediatrics and Child Health, Kurume University School of Medicine, Fukuoka, Japan.

2. Kurume University School of Nursing, Fukuoka, Japan.

3. Dept of Obstetrics and Gynaecology, Kurume University School of Medicine, Fukuoka, Japan.

4. Dept of Obstetrics and Gynaecology, Kyushu University School of Medicine, Fukuoka, Japan.

**Correspondence to:** Dr Osuke Iwata

Centre for Cognitive & Developmental Neuroscience, Kurume University School of Medicine

67 Asahimachi, Kurume, Fukuoka, 830-0011 Japan

E-mail: o.iwata@ucl.ac.uk Tel: +81 942 31-7565 Fax: +81 942 38-1792

**Online Supplemental Table 1: Control variables of nighttime sleep durations for mothers**

|  |  | β | 95% confidence interval | | p |
| --- | --- | --- | --- | --- | --- |
|  |  | Average | Lower | Upper |  |
| Univariate analysis | |  |  |  |  |
| Season |  |  |  |  |  |
|  | Winter | 1.01 | 0.78 | 1.31 | 0.948 |
|  | Spring | 1.18 | 0.93 | 1.48 | 0.168 |
|  | Summer | 0.94 | 0.73 | 1.21 | 0.638 |
|  | Autumn | Reference | | | |
| Sleep onset time for mothers | |  |  |  |  |
|  | Regularly ≤ 23:00 h | 2.83 | 2.34 | 3.42 | <0.001 |
|  | Regularly > 23:00 h | 1.49 | 1.19 | 1.85 | <0.001 |
|  | Irregular | Reference | | | |
| Daytime nap duration (h) | | 0.996 | 0.994 | 0.998 | <0.001 |
| Infant’s nighttime sleep duration (h) | | 1.37 | 1.31 | 1.44 | <0.001 |
|  |  |  |  |  |  |
| Multivariate analysis | |  |  |  |  |
| Sleep onset time for mothers | |  |  |  |  |
|  | Regularly ≤ 23:00 h | 2.30 | 1.92 | 2.77 | <0.001 |
|  | Regularly > 23:00 h | 1.36 | 1.10 | 1.68 | 0.005 |
|  | Irregular | Reference | | | |
| Daytime nap duration (h) | | 0.998 | 0.996 | 1.00 | 0.091 |
| Infant’s nighttime sleep duration (h) | | 1.31 | 1.25 | 1.37 | <0.001 |

Independent variables for maternal nighttime sleep duration were assessed using a generalised linear model. Statistical findings are presented without correction for multiple comparisons.

**Online Supplemental Text 1: Sleep questionnaire for mothers of newborn infants**

This questionnaire was developed and conducted in Japanese, and is presented after translation into English for the readers’ convenience.

**Section 1: Please answer the questions in relation to you and your family**

1. How old are you?

□□ years old

2. What is your main occupation at present (before maternity leave or temporary/permanent retirement)?

Please specify:

3. Does your main occupation involve night-shift work?

□ no □ yes

4. How old is your spouse/partner?

□□ years old

5. What is the main occupation of your spouse/partner at present?

Please specify:

6. Does the main occupation of your spouse/partner involve night-shift work?

□ no □ yes

7. What is your highest educational attainment?

□ university or college □ high school □ junior high school

8. Are you satisfied with the current income of your family?

□ fully satisfied □ generally satisfied □ dissatisfied

9. Tick the members in your household currently living together within the same property.

□ my spouse/partner □ my father (□ in law) □ my mother (□ in law)

□ my child/children

(Please specify the number and age of the child/children: )

□ other (Please specify: )

**Section 2: Please tick/enter the following information about the birth of your baby.**

Date and time of birth: month □□ day □□ time □□h: □□min.

Gestational age: □□ weeks and □ day(s)

Delivery mode: □ vaginal □ elective caesarean □ emergency caesarean

Body weight: at birth □□□□g

at discharge □□□□g on day □□

today □□□□g on day □□

**Section 3: Please answer the questions about your other baby’s caregivers.**

1. Do you have an alternative person(s) who can take care of your baby?

□ no □ yes

Please answer questions 2 to 4 only if your answer to question 1 was “yes.”

2. Please specify the relationship with the alternative caregiver(s).

3. How satisfied are you with the alternative caregiver’s support?

□ fully satisfied □ generally satisfied □ a little dissatisfied □ dissatisfied

4. What kind of support does the alternative caregiver provide to you and your baby?

□ feeding □ bathing □ nappy change □ cradle □ housekeeping duties

□ other (Please specify: )

**Section 4: Please answer the following questions about your baby’s typical sleep pattern and its environment over the last 7 days.**

1. How long does your baby spend asleep during the day (8:00 to 20:00)?

□□h: □□min.

2. How frequently does your baby typically wake up during the day (8:00 to 20:00)?

every □□h: □□min.

3. How long does your baby spend asleep during the night (20:00 to 8:00)?

□□h: □□min.

3. How frequently does your baby wake up during the night (20:00 to 8:00)?

every □□h: □□min.

4. Do you share your bedroom with your baby?

□ no □ yes

If yes, do you share your bed or sleeping mattress with your baby?

□ no □ yes

5. How quiet is the bedroom?

□ quiet □ relatively quiet □ noisy

6. How bright is the bedroom?

□ bright □ relatively bright □ dim

7. How regular is the light-off time for your baby?

□ regular (Please specify the time: □□h: □□min.)

□ relatively regular (Please specify the time: □□h: □□min.)

□ no regular light-off time

8. Do you have a routine when your baby cries during the night?

□ no □ yes

Please answer questions 9 to 10 only if your answer to question 8 was “yes.”

9. Please tick all that apply regarding your routine when your baby cries during the night:

□ feeding □ handling/cradling □ other (Please specify: )

10. How long do you continue the routine described in the answer to question 9?

□ until my baby gets calm or falls asleep □ only for a short period

11. What milk do you feed your baby?

□ breast milk only □ mainly breast milk □ mainly formula □ formula only

12. How frequently do you feed your baby during the day (8:00 to 20:00)?

every □□h: □□min.

13. How frequently do you feed your baby during the night (20:00 to 8:00)?

every □□h: □□min.

14. Do you have any concerns or worries about your baby? Please tick any that apply.

□ eczema □ respiration □ vomiting □ constipation/diarrhoea

□ weight gain □ crying □ feeding □ sleep

□ motor development □ cognitive development

□ other (Please specify: )

15. How confident or anxious are you about caring for your baby?

□ very confident □ relatively confident □ relatively anxious □ very anxious

**Section 5: Please answer the questions about your sleep condition during the last month of your pregnancy.**

1. How regular was your bed time?

□ regular (Please specify the time: □□h: □□min.)

□ relatively regular (Please specify the time: □□h: □□min.)

□ no regular bed time

2. How long did it take you to get to sleep?

□□min.

3. How frequent was your nocturnal awakening after the onset of sleep?

□□ times

4. What time was your typical wake-up time?

□□h: □□min.

5. How long was your typical nighttime sleep duration (20:00 to 8:00)?

□□h: □□min.

6. Did you have a daytime nap?

□ no □ yes (If “yes,” please specify for how long: □□h: □□min.)

7. How satisfied were you with your sleep?

□ fully satisfied □ mildly dissatisfied

□ dissatisfied, but no difficulty getting through the day □ dissatisfied, with difficulty getting through the day

**Section 6: Please answer the questions about your sleep condition over the last 7 days.**

1. How regular is your bed time?

□ regular (Please specify the time: □□h: □□min.)

□ relatively regular (Please specify the time: □□h: □□min.)

□ no regular bed time

2. How long does it typically take you to get to sleep?

□□min.

3. How frequent is your nocturnal awakening after the onset of sleep?

□□ times

4. What time is your typical wake-up time?

□□h: □□min.

5. How long is your typical nighttime sleep duration (20:00 to 8:00)?

□□h: □□min.

6. Do you have a daytime nap?

□ no □ yes (If “yes,” please specify for how long: □□h: □□min.)

7 How satisfied are you with your sleep?

□ fully satisfied □ mildly dissatisfied

□ dissatisfied, but no difficulty in getting through the day □ dissatisfied, with difficulty getting through the day

**Section 7: Please answer the questions regarding sleepiness during the day.**

(Eight questions from the Epworth Sleepiness Scale by Johns. The Japanese translation is by Fukuhara.)

**Section 8: Please answer the questions regarding your emotional condition.**

(Ten questions from Edinburgh Postnatal Depression Scale by Cox et al. The Japanese translation is by Okano.)
